# Supplementary material for: Characterization of the Complete Mitochondrial Genome of Drabescus ineffectus and Roxasellana stellata (Hemiptera: Cicadellidae: Deltocephalinae: Drabescini) and Their Phylogenetic Implications
Source: Insects. 2020 Aug 14;11(8):534. doi: 10.3390/insects11080534 (PMC7469207; doi:10.3390/insects11080534)
Supplement: Supplementary file 1 [file insects-11-00534-s001.pdf]

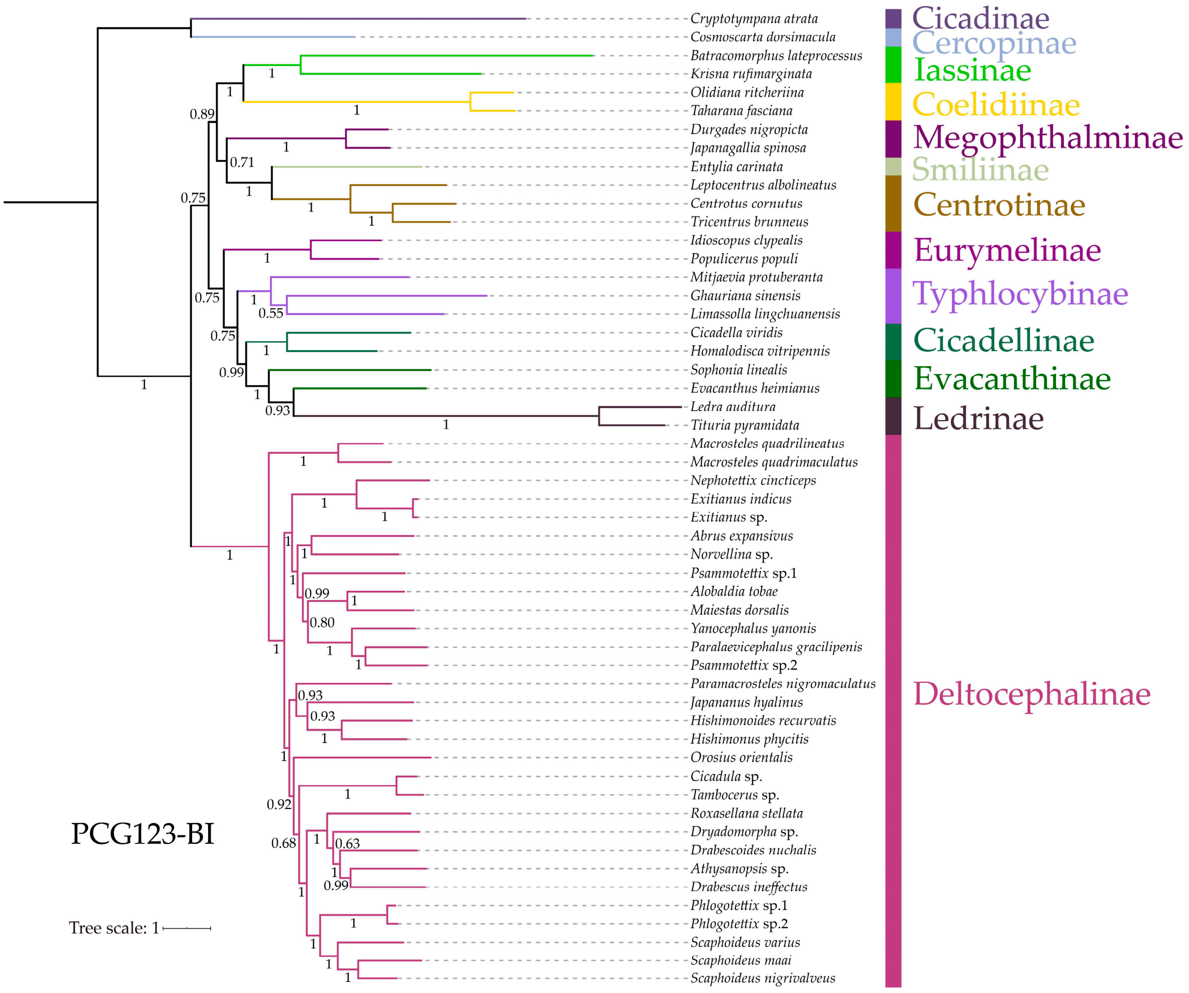

Figure S1. Phylogenetic tree inferred from BI method based on PCG123 dataset. Numbers on branches are posterior probabilities (PP).

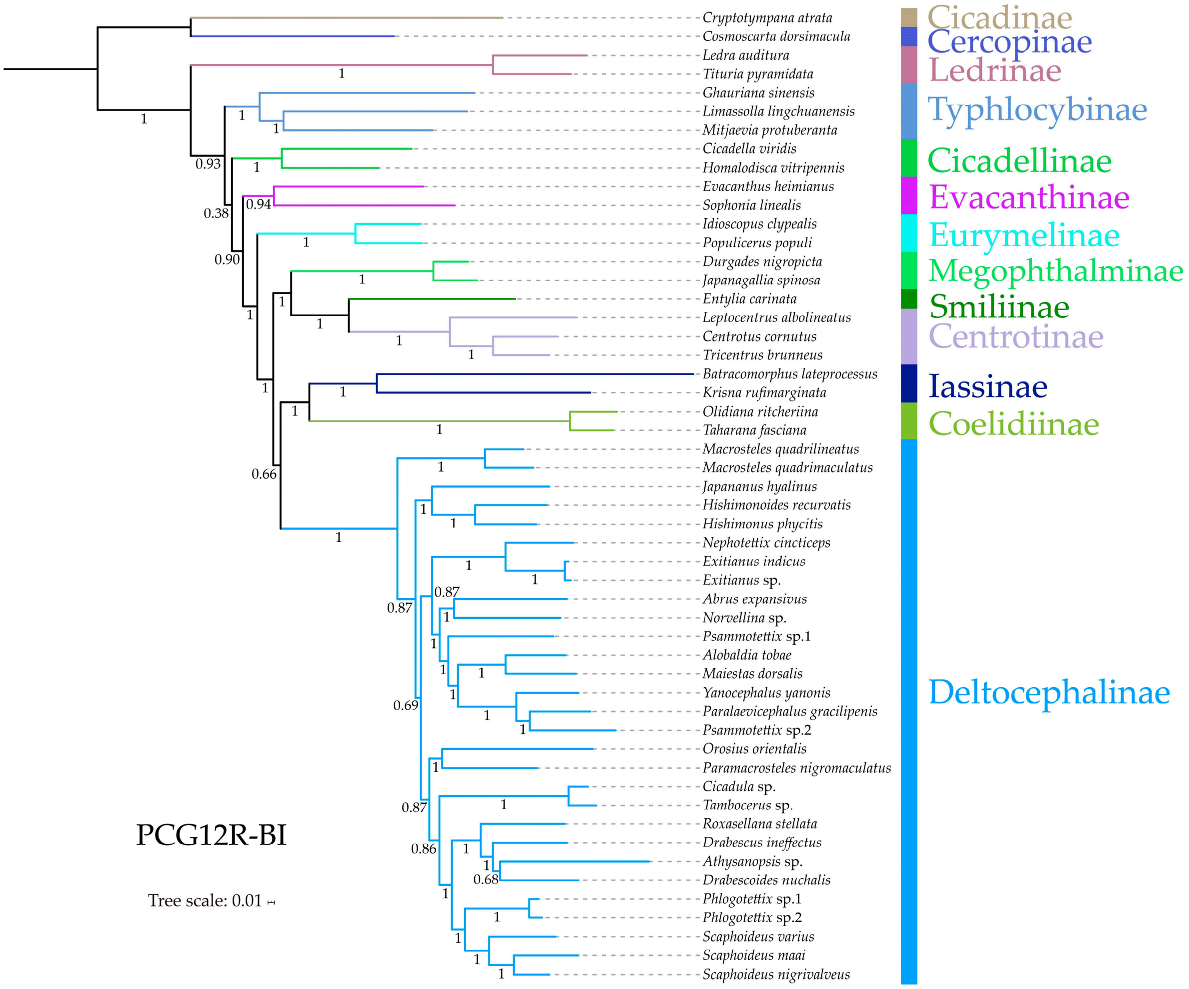

Figure S2. Phylogenetic tree inferred from BI method based on PCG12R dataset. Numbers on branches are posterior probabilities (PP).

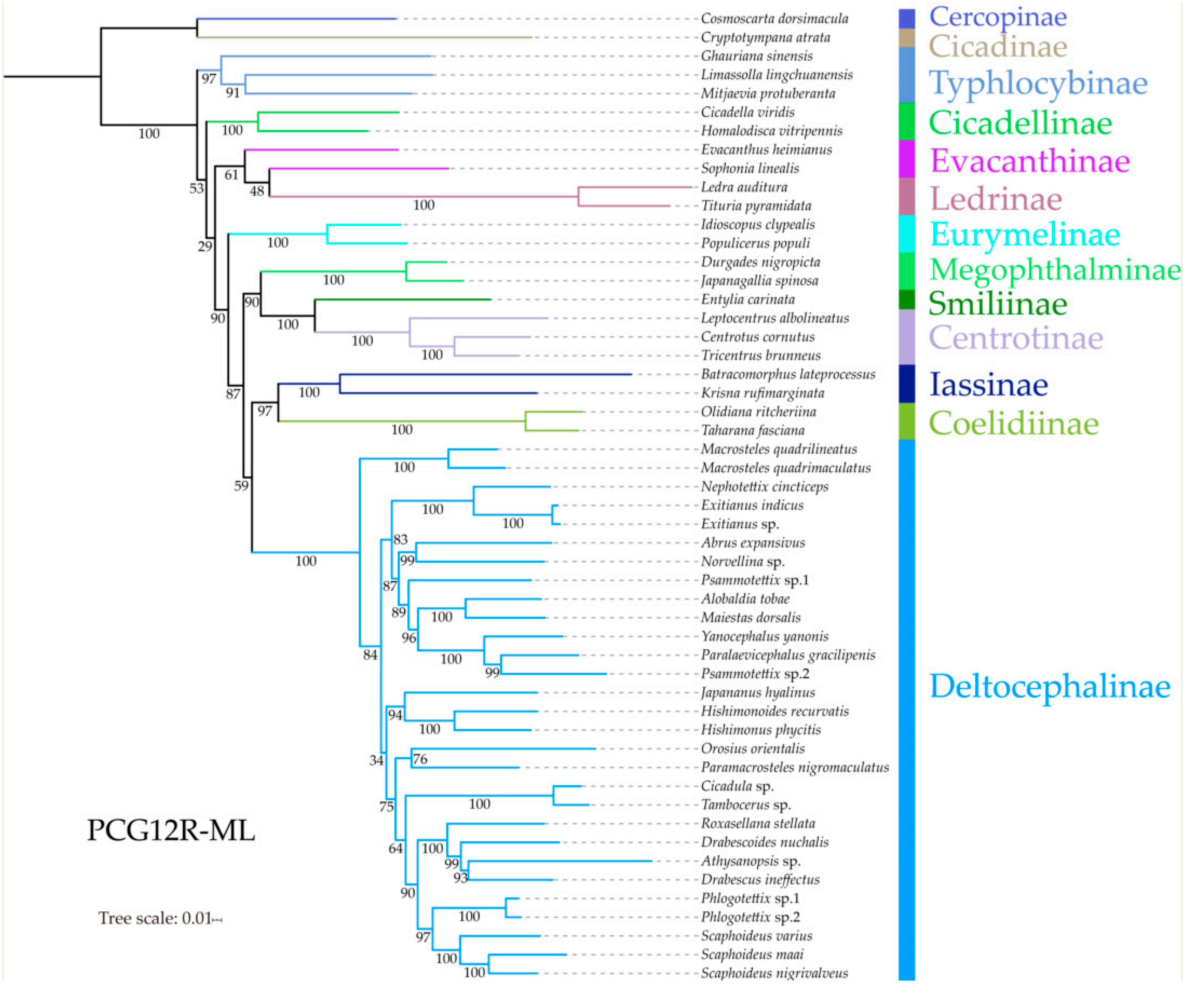

Figure S3. Phylogenetic tree inferred from ML method based on PCG12R dataset. Numbers on branches are bootstrap support values (BS).

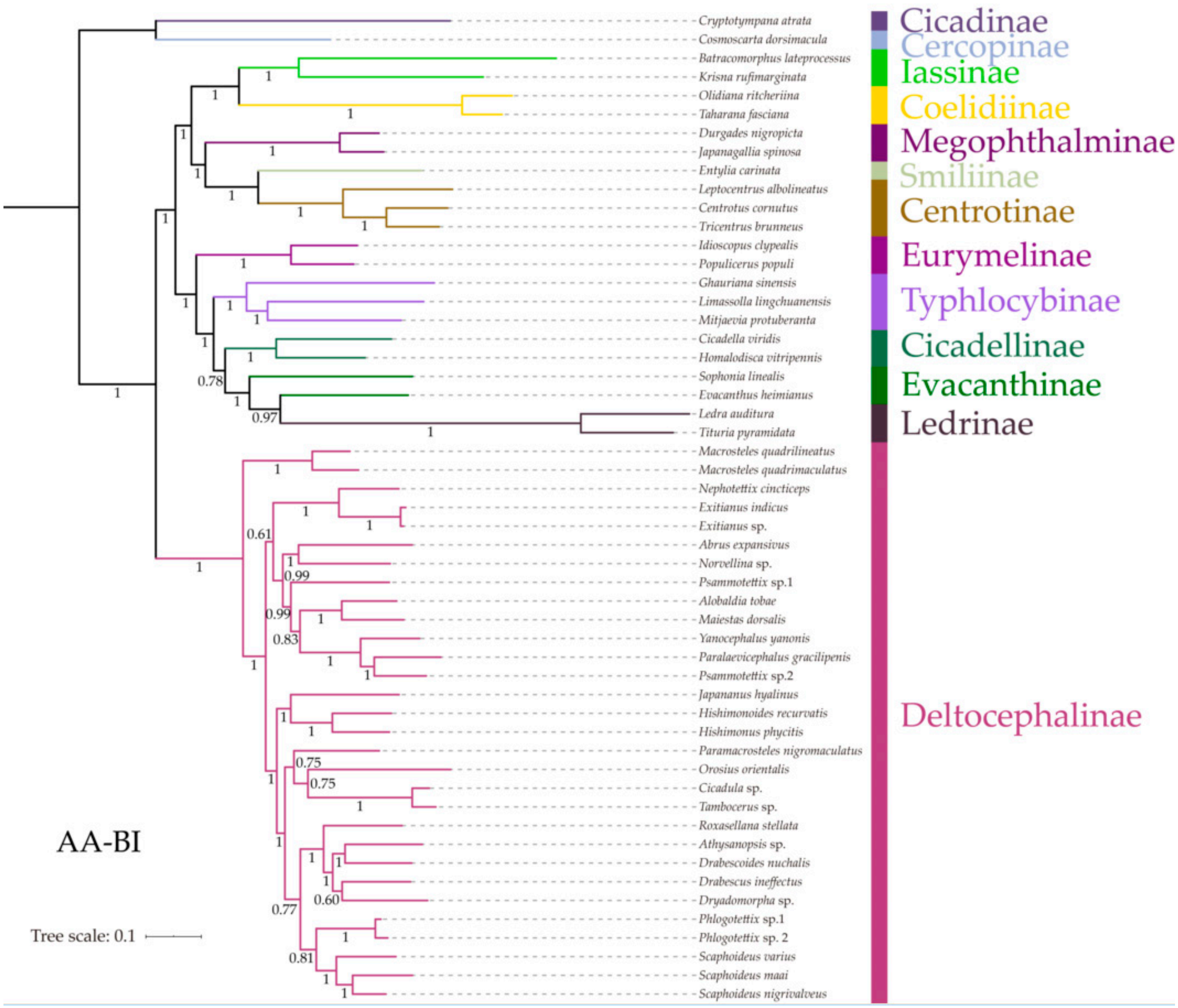

Figure S4. Phylogenetic tree inferred from BI method based on amino acid sequences. Numbers on branches are posterior probabilities (PP).

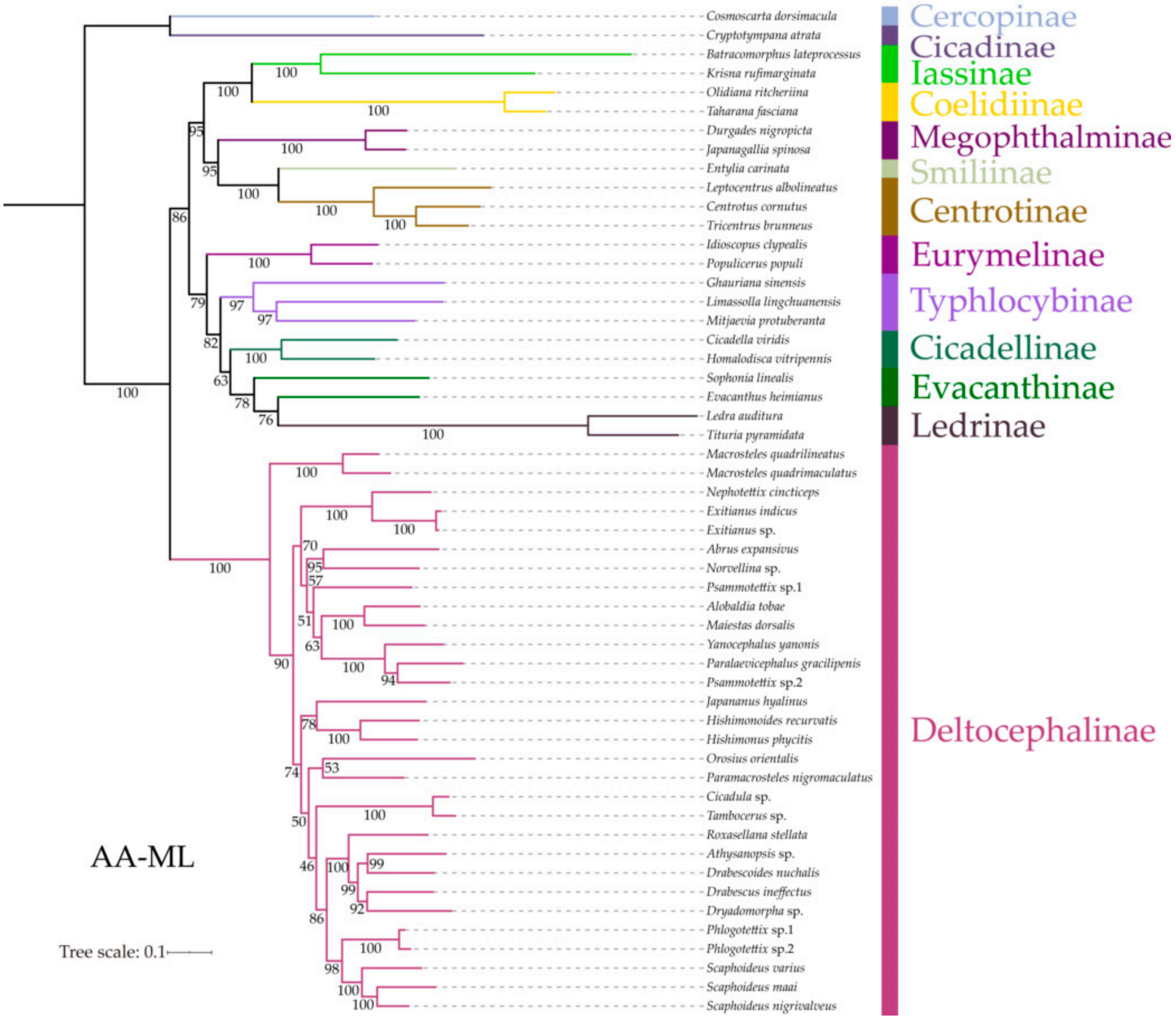

Figure S5. Phylogenetic tree inferred from ML method based on amino acid sequences. Numbers on branches are bootstrap support values (BS).

Table S1. Mitogenomic organization of *Drabescus ineffectus* and *Roxasellana stellata*.

Gene.	Position		Size (bp)	Intergenic Nucleotides	Codon		Strand
	From	To			Start	Stop	
trnI	1 / 1	66 / 70	66 / 70				J / J
trnQ	64 / 68	132 / 136	69 / 69	−3 / −3			N / N
trnM	142 / 143	206 / 206	65 / 64	9 / 6			J / J
nad2	207 / 207	1178 / 1178	972 / 972	0 / 0	ATG/ATG	TAA / TAA	J / J
trnW	1177 / 1177	1244 / 1249	68 / 73	−2 / −2			J / J
trnC	1237 / 1242	1299 / 1309	63 / 68	−8 / −8			N / N
trnY	1319 / 1325	1381 / 1386	63 / 62	19 / 15			N / N
cox1	1402 / 1404	2937 / 2939	1536 / 1536	20 / 17	ATG / ATG	TAA / TAA	J / J
trnL2	2943 / 2944	3005 / 3005	63 / 62	5 / 4			J / J
cox2	3006 / 3006	3690 / 3687	685 / 682	0 / 0	ATA / ATA	T / T	J / J
trnK	3691 / 3688	3760 / 3758	70 / 71	0 / 0			J / J
trnD	3761 / 3768	3829 / 3829	69 / 62	0 / 9			J / J
atp8	3830 / 3830	3982 / 3982	153 / 153	0 / 0	ATA / ATC	TAA / TAA	J / J
atp6	3976 / 3976	4629 / 4629	654 / 654	−7 / −7	ATG / ATG	TAA / TAA	J / J
cox3	4631 / 4632	5410 / 5414	780/783	1 / 2	ATG / ATG	TAG / TAA	J / J
trnG	5413 / 5424	5476 / 5485	64 / 62	2 / 9			J / J
nad3	5477 / 5486	5830 / 5839	354 / 354	0 / 0	ATT / ATT	TAG / TAA	J / J
trnA	5830 / 5845	5891 / 5905	62 / 61	−1 / 5			J / J
trnR	5895 / 5906	5956 / 5969	62 / 64	3 / 0			J / J
trnN	5956 / 5970	6020 / 6035	65 / 66	−1 / 0			J / J
trnS1	6020 / 6035	6086 / 6100	67 / 66	−1 / −1			J / J
trnE	6089 / 6098	6154 / 6160	66 / 63	2 / −3			J / J
trnF	6153 / 6161	6219 / 6224	67 / 64	−2 / 0			N / N
nad5	6221 / 6227	7897 / 7900	1677 / 1674	1 / 2	ATT / TTG	TAA / TAA	N / N
trnH	7895 / 7901	7957 / 7965	63 / 65	-3 / 0			N / N
nad4	7958 / 7967	9269 / 9275	1312 / 1309	0 / 1	ATG / ATG	T / T	N / N
nad4L	9263 / 9269	9538 / 9544	276 / 276	−7 / −7	ATA / ATT	TAA / TAA	N / N
trnT	9541 / 9547	9604 / 9610	64 / 64	2 / 2			J / J
trnP	9605 / 9611	9671 / 9677	67 / 67	0 / 0			N / N

nad6	9674 / 9680	10162 / 10156	489 / 477	2 / 2	ATT / ATA	TAA / TAA	J / J
cytb	10155 / 10160	11291 / 11296	1137 / 1137	−8 / 3	ATG / ATG	TAA / TAA	J / J
trnS2	11292 / 11296	11358 / 11359	67 / 64	0 / −1			J / J
nad1	11361 / 11364	12293 / 12291	933 / 928	2 / 4	ATT / ATA	TAG / T	N / N
trnL1	12294 / 12292	12357 / 12358	64 / 67	0 / 0			N / N
rrnL	12358 / 12359	13577 / 13568	1220 / 1210	0 / 0			N / N
trnV	13578 / 13569	13642 / 13635	65 / 67	0 / 0			N / N
rrnS	13643 / 13636	14363 / 14378	721/ 743	0 / 0			N / N
CR	14364 / 14379	15744 / 15361	1381 / 983	0 / 0			

Table S2. Nucleotide composition of the mitogenomes of *Drabescus ineffectus* and *Roxasellana stellata*.

Feature	Size (bp)	A%	C%	G%	T%	AT%	GC %	AT-skew	GC-skew
				Drabescus ineffectus					
Whole genome	15744	41.7	13	9.9	35.4	77.1	22.9	0.081	−0.133
PCGs	10956	33	12.2	12.4	42.4	75.4	24.6	−0.124	0.009
1st codon position	3652	36.8	11.4	16.7	35	71.9	28.1	0.024	0.187
2nd codon position	3652	20.7	17.6	13.7	48	68.7	31.3	−0.397	−0.126
3rd codon position	3652	41.6	7.4	6.8	44.2	85.8	14.2	−0.030	−0.048
tRNAs	1439	39.3	9.2	13.4	38.2	77.4	22.6	0.014	0.188
rRNAs	1941	35.2	7.5	12.5	44.8	80	20	−0.120	0.247
CR	1381	41.8	7.7	7.0	43.5	85.3	14.7	−0.020	−0.054
				Roxasellana stellata					
Whole genome	15361	41.4	14	9.9	34.6	76	24	0.089	−0.17
PCGs	10932	33.9	12.5	12.2	41.4	75.3	24.7	−0.099	−0.013
1st codon position	3644	36.9	11.4	16.5	35.3	72.1	27.9	0.022	0.184
2nd codon position	3644	21	17.3	14	47.7	68.7	31.3	−0.39	−0.105
3rd codon position	3644	43.9	8.9	6.1	41	84.9	15.1	0.034	−0.185
tRNAs	1441	38.6	10	13.5	37.9	76.5	23.5	0.009	0.15
rRNAs	1953	36.3	7.2	12.4	44.1	80.4	19.6	−0.098	0.264
CR	983	38.3	12	13.2	36.2	74.7	25.3	0.027	0.048

Table S3. Nucleotide compositions in regions of the Drabescini mitochondrial genomes.

Species	Whole genome		AT-skew	GC-skew	PCGs		tRNAs		rRNAs		CR	
	Size(bp)	AT %			Size(bp)	AT %	Size(bp)	AT %	Size(bp)	AT %	Size(bp)	AT %
Drabescoides nuchalis	15309	75.6	0.099	−0.175	10932	74.6	1480	77.1	1937	79	956	77.3
Drabescus ineffectus	15744	77.1	0.081	−0.133	10956	75.4	1439	77.4	1941	80	1381	85.3
Roxasellana stellata	15361	76	0.089	−0.17	10932	75.3	1441	76.5	1953	80.4	983	74.7
Athysanopsis sp.	14573	73.6	0.134	−0.219	10938	73.2	1446	75.1	1821	78.3	200	73.5
Dryadomorpha sp.	12297	73.4	0.109	−0.117	10656	73.7	1304	74.2	–	–	240	87.5

Table S4. Best partitioning scheme and nucleotide substitution models for different datasets selected by PartitionFinder.

Data Matrix	Subset Partitions	Model
PCG123-BI	1. *atp6*_pos1	GTR + I + G
	2. *nad6*_pos2, *nad3*_pos2, *atp6*_pos2, *atp8*_pos2, *nad2*_pos2	GTR + I + G
	3. *cox3*_pos3, *cytb*_pos3, *nad6*_pos3, *nad3*_pos3, *atp8*_pos3, *cox2*_pos3, *atp6*_pos3	GTR + I + G
	4. *atp8*_pos1, *nad6*_pos1, *nad2*_pos1, *nad3*_pos1	GTR + I + G
	5. *cytb*_pos1, *cox1*_pos1	GTR + I + G
	6. *cox1*_pos2, *cox3*_pos2, *cox2*_pos2, *cytb*_pos2	GTR + I + G
	7. *cox1*_pos3	HKY + I + G
	8. *nad1*_pos1, *cox3*_pos1, *cox2*_pos1	GTR + I + G
	9. *nad1*_pos2, *nad4*_pos2, *nad4L*_pos2, *nad5*_pos2	GTR + I + G
	10. *nad1*_pos3	HKY + I + G
	11. *nad2*_pos3	GTR + G
	12. *nad4L*_pos1, *nad4*_pos1, *nad5*_pos1	GTR + I + G
	13. *nad4*_pos3, *nad5*_pos3, *nad4L*_pos3	HKY + I + G
PCG123-ML	1. *atp6*_pos1	GTR + I + G
	2. *cox2*_pos2, *atp6*_pos2, *cytb*_pos2, *cox3*_pos2	TVM + I + G
	3. *nad3*_pos3, *atp8*_pos3, *atp6*_pos3, *cox1*_pos3, *cox2*_pos3	K81UF + I + G
	4. *atp8*_pos1, *nad6*_pos1, *nad2*_pos1, *nad3*_pos1	GTR + I + G
	5. *atp8*_pos2, *nad3*_pos2, *nad2*_pos2, *nad6*_pos2	TVM + I + G
	6. *cytb*_pos1, *cox1*_pos1	GTR + I + G
	7. *cox1*_pos2	TVM + I + G
	8. *nad1*_pos1, *cox3*_pos1, *cox2*_pos1	GTR + I + G
	9. *cox3*_pos3, *cytb*_pos3, *nad6*_pos3	GTR + G
	10. *nad1*_pos2, *nad4*_pos2, *nad4L*_pos2, *nad5*_pos2	GTR + I + G
	11. *nad1*_pos3, *nad4*_pos3, *nad5*_pos3, *nad4L*_pos3	TIM + I + G
	12. *nad2*_pos3	TRN + G
	13. *nad4*_pos1, *nad5*_pos1, *nad4L*_pos1	GTR + I + G
PCG12R-BI	1. *nad3*, *atp6*, *atp8*, *nad6*, nad2	GTR + I + G
	2. *cox1*	GTR + I + G
	3. *cytb*, *cox2*, *cox3*	GTR + I + G
	4. *nad1*, *nad4*, *nad4L*, *nad5*	GTR + I + G

	5. *rrnL*, *rrnS*	GTR + I + G
PCG12R-ML	1. *nad2*, *nad6*, *nad3*, *apt6*	GTR + I + G
	2. *atp8*	TRN + I + G
	3. *cox1*	GTR + I + G
	4. *cytb*, *cox3*, *cox2*	GTR + I + G
	5. *nad1*, *nad4*, *nad5*, *nad4L*	TVM + I + G
	6. *rrnL*, *rrnS*	GTR + I + G
AA-BI	1. *cytb*, *nad1*, *cox2*, *atp6*, *cox3*, *atp8*	MTREV + I + G
	2. *cox1*	MTREV + I + G
	3. *nad2*	MTREV+ I + G
	4. *nad6*, *nad3*, *nad4L*	MTREV + I + G
	5. *nad4*, *nad5*	MTREV + I + G
AA-ML	1. *atp8*, *cox2*, *cytb*, *nad6*, *nad3*, *atp6*, *nad2*, *cox3*	MTART + I + G + F
	2. *cox1*	MTART + I + G
	3. *nad4l*, *nad1*, *nad5*, *nad4*	MTART + I + G + F
